# Validation of the RayStation Monte Carlo dose calculation algorithm using a realistic lung phantom

**DOI:** 10.1002/acm2.12777

**Published:** 2019-11-25

**Authors:** Andries N. Schreuder, Daniel S. Bridges, Lauren Rigsby, Marc Blakey, Martin Janson, Samantha G. Hedrick, John B. Wilkinson

**Affiliations:** ^1^ Provision Center for Proton Therapy – Knoxville 6450 Provision Cares Way Knoxville TN 37909 USA; ^2^ RaySearch Laboratories Sveavägen 44 SE‐103 65 Stockholm Sweden

**Keywords:** charged particle therapy, lung, Monte Carlo, pencil beam scanning, pencil‐beam algorithm, radiation

## Abstract

**Purpose:**

Our purposes are to compare the accuracy of RaySearch's analytical pencil beam (APB) and Monte Carlo (MC) algorithms for clinical proton therapy and to present clinical validation data using a novel animal tissue lung phantom.

**Methods:**

We constructed a realistic lung phantom composed of a rack of lamb resting on a stack of rectangular natural cork slabs simulating lung tissue. The tumor was simulated using 70% lean ground lamb meat inserted in a spherical hole with diameter 40 ± 5 mm carved into the cork slabs. A single‐field plan using an anterior beam and a two‐field plan using two anterior‐oblique beams were delivered to the phantom. Ion chamber array measurements were taken medial and distal to the tumor. Measured doses were compared with calculated RayStation APB and MC calculated doses.

**Results:**

Our lung phantom enabled measurements with the MatriXX PT at multiple depths in the phantom. Using the MC calculations, the 3%/3 mm gamma index pass rates, comparing measured with calculated doses, for the distal planes were 74.5% and 85.3% for the APB and 99.1% and 92% for the MC algorithms. The measured data revealed up to 46% and 30% underdosing within the distal regions of the target volume for the single and the two field plans when APB calculations are used. These discrepancies reduced to less than 18% and 7% respectively using the MC calculations.

**Conclusions:**

RaySearch Laboratories' Monte Carlo dose calculation algorithm is superior to the pencil‐beam algorithm for lung targets. Clinicians relying on the analytical pencil‐beam algorithm should be aware of its pitfalls for this site and verify dose prior to delivery. We conclude that the RayStation MC algorithm is reliable and more accurate than the APB algorithm for lung targets and therefore should be used to plan proton therapy for patients with lung cancer.

## 1 INTRODUCTION

Lung and bronchus is now the most common cancer site in 16 states and second overall in the United States, with only 12% fewer estimated cases than female breast.^1^ Given the principle to keep radiation exposure as low as reasonably achievable,^2^ combined with the benefits of hypo fractionated proton therapy,^3^, ^4^, ^5^, ^6^ the need to increase the accuracy of proton radiotherapy to thoracic sites has thus become imperative. Most proton clinics are currently using commercially available analytical pencil beam (APB) algorithms.^7^, ^8^, ^9^ These APB algorithms are typically designed to be computationally efficient, but inherently include simplifications of the transport problem as compared to, for example, Monte Carlo (MC)‐based algorithms. Consequently, the APB algorithm sometimes suffers loss of accuracy in areas of inhomogeneity, such as in lung, where even small algorithmic deficiencies can result in significant shifts of dose distributions.^10^ It has been shown by Taylor et al. that the APB algorithm is “doing a poor job” of predicting dose in lung tumors, with over‐predictions up to 46% in the PTV.^11^ In the case of lateral heterogeneities, MC dose calculation algorithms are superior in calculating accurate dose distributions.^12^, ^13^, ^14^ MC is viewed as the gold standard for dose calculation for most radiation transport calculations, but traditional MC algorithms such as MCNPX^15^, ^16^ and Geant4^16^ are too slow to keep up with clinical workflow. To combat this problem, Fast Monte Carlo (FMC) dose engines have been developed for clinical use.^10^, ^17^ A study by Sorriaux et al. compared both MC and FMC dose engines with APB in clinical situations of heterogeneity.^18^ They found that FMC corresponded well with data measured in an inhomogeneous phantom made of water surrounding a long insert of bone tissue substitute, whereas more than half of the APB dose distributions failed gamma‐index analysis.^16^

We explain in another study how the RaySearch APB and MC algorithms work.^19^ When the MC dose engine became available in RayStation 6 (RaySearch Laboratories AB, Stockholm, Sweden), we proposed to validate it using animal tissue and realistic phantoms, because the use of animal tissue phantoms in dose validation has seen useful results,^20^, ^21^ similar to using wood or cork to approximate lung tissue.^22^, ^23^ Previously, we studied these algorithms applied to animal neck phantoms and a water‐based breast phantom.^19^ In this study, we focused on validating the MC algorithm for a more complex lung phantom made of a composite of lamb ribs, ground lamb meat and cork. We demonstrate that we can measure dose inside a realistic lung tumor phantom for a target *not* adjacent to the chest wall, that is, a tumor surrounded by lower density lung tissue. We then compared the measured data with doses calculated using the RaySearch APB and MC algorithms. We searched articles available to the public from multiple journals concerning lung phantoms. Although a number of phantoms have been tested, including solid water,^24^, ^25^ cork and solid water,^26^, ^27^, ^28^, ^29^ balsa wood and solid water,^30^ balsa wood and cork,^11^ cork and plastic,^31^, ^32^ cork and acrylic,^33^, ^34^ polystyrene and cork,^35^ bolus and sponge,^36^ foam,^37^, ^38^ and even wood,^39^, ^40^ it appears that we are the first to report open‐access on the use of a cork‐and‐animal‐tissue lung tumor phantom.

## 2 METHODS

### 2.1 Dose validation phantom

**Figure 1:**
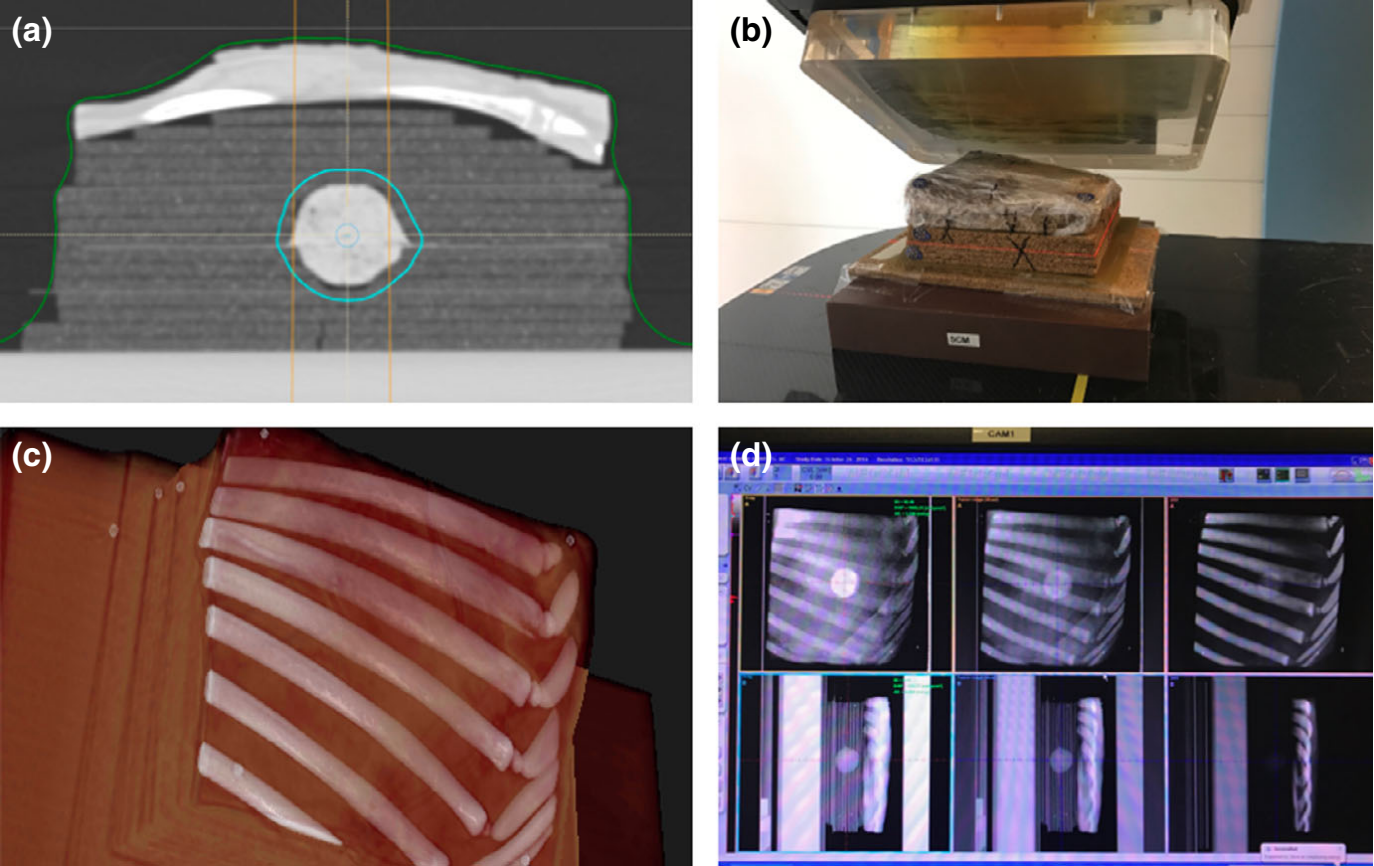
A CT image of the realistic lung phantom (panel a) placed on solid water in the position to be irradiated (panel b) for the left anterior oblique beam of the two‐field lung plan. Panel c shows a 3 D rendering from the CT data illustrating the ribs in the beam path. Panel d shows the orthogonal x rays and Digital Reconstructed Radiographs (DRRs) used for positioning the phantom using the VeriSuite IGRT System.

The lung phantom is shown in Fig. 1. The lung phantom was composed of a rack of lamb containing real rib bones, intercostal muscle, and fat. To simulate lung tissue beneath the lamb rack, we placed 5.0 ± 0.5 mm thick layers of Quartet cork (ACCO Brands, Lake Zurich, Illinois; SKU 48112Q) obtained from a hardware store. The cork slabs and the rack of lamb were pinned together using wooden toothpicks to allow for splitting and re‐assembling the phantom accurately enabling measurements “inside” the phantom.

An approximately spherical hole 40 ± 5 mm in diameter was cut into the cork after five cork slabs. This placed the proximal edge of the cavity at a physical depth of approximately 2.5 cm beyond the chest wall. The cavity was filled with 70% lean ground lamb meat to simulate a solid lung tumor with an effective mass density slightly less than muscle. We used plastic Glad® Cling Wrap to divide this tumor in two halves so that planar dose measurements could be made within and distal to the tumor. The physical thickness of the simulated chest wall anterior to the tumor varied between 1.5 and 1.8 cm. The gross tumor volume (GTV), represented by the cavity filled with ground lamb meat and drawn on the planning CT scan, had a volume of 26.3 cm^3^ which is representative of solid lung tumors our clinic treats with protons. Smaller lung tumors are typically treated with x rays using SBRT techniques. The average HU of the GTV was 46 ± 21. The entire phantom was constructed with the rack of lamb frozen to measure the geometry, cut the cork slabs, and carve the cavity for the tumor in the cork. On the day of the experiment, the rack of lamb and ground lamb meat were thawed, and the latter was inserted into the cavity. In a single day, the phantom was scanned, treatment planning was performed, the beams were delivered to the phantom, and the doses were measured.

### 2.2 Treatment planning

**Figure 2:**
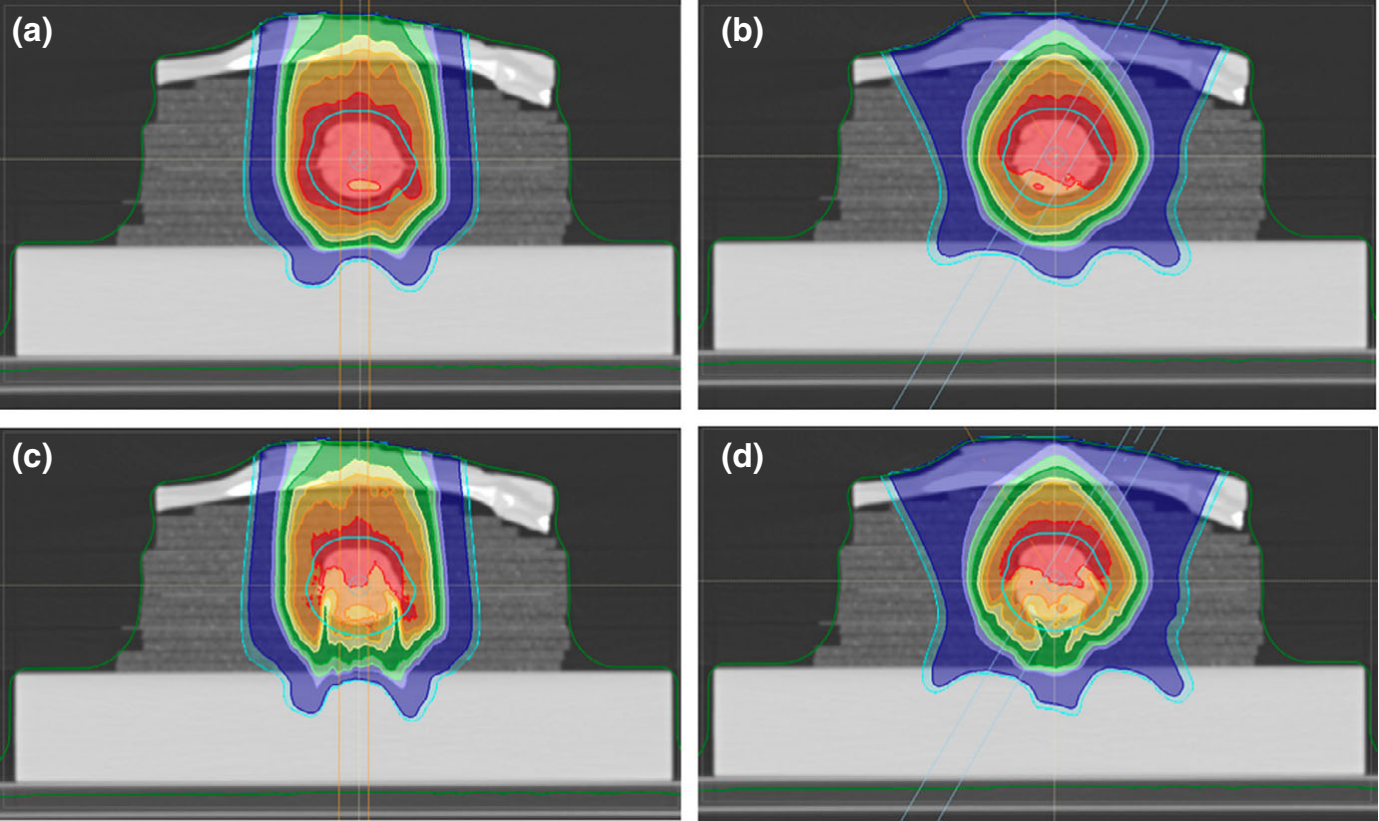
Dose distributions for the two lung phantom plans shown in the axial CT slices through the isocenter. The 1 Field lung phantom plan dose distribution calculated with APB is shown in panel a and the corresponding MC dose distribution is shown in panel c. The 2 Field lung phantom plan dose distribution calculated with APB is shown in panel b and the corresponding MC dose distribution is shown in panel d. APB, analytical pencil beam.

The lung phantom was scanned on a Siemens Somatom Definition AS CT scanner (Siemens Medical Solutions USA, Inc., Malvern, PA, USA) using a 50 cm Field of View (FOV) obtaining 512 × 512 pixel axial images (0.98 × 0.98 mm^2^ pixel size) reconstructed with a 4 mm slice distance between images (131 axial images). The phantom was marked with a pen and BBs to ensure the alignment could be accurately reproduced. Using RayStation, the GTV was delineated and expanded to a CTV using a 5 mm uniform margin. The CTV was used as the target in the optimization of the plans. We developed two pencil‐beam scanning (PBS) plans as shown in Fig. 2. The first plan used a single anterior field and a second plan used two anterior oblique fields that were optimized using the single field uniform dose (SFUD) technique i.e. each field delivered a uniform dose to the CTV. For both plans, the isocenter was centered within the tumor. A 7.5 cm WET Lucite range shifter with physical thickness of 6.7 cm was used, and the airgap, here defined as the smallest distance between the range shifter and the phantom surface, ‐was kept as small as possible. An air gap of 1.6 cm was used for the single‐field lung plan. Beam 1 of the two‐field plan also used a 1.6 cm airgap while beam 2 used a 2 cm airgap. The two plans were optimized to deliver a uniform dose of 2 Gy(RBE) in one fraction to the target.

The plans were optimized using the APB algorithm in RayStation (RS) 6.0 using a 1 × 1 × 1 mm^3^ calculation grid. The doses of the APB plans were then recomputed using the RS 6.0 MC dose engine to a statistical uncertainty in the high dose region of 0.5% per beam. The RS6.0 APB doses were subsequently recomputed using RS 6.2, due to an updated handling of the range shifter in the APB algorithm that became available in that version.

**Figure 3:**
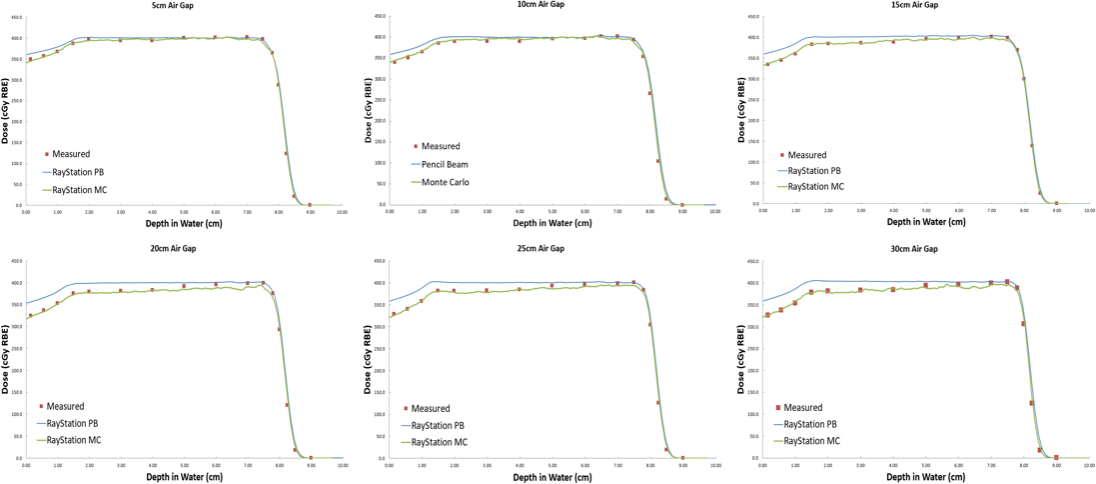
A comparison between measured and calculated central axis (CAX) doses for a PBS beam for different air gaps between the range shifter and the water surface. The measured data points are indicated by the red squares while the MC data and the APB calculated doses are shown by the green and blue lines respectively. The extent of the airgap for each graph is printed as the title of each panel. APB, analytical pencil beam.

One of the known deficiencies of the APB algorithm is calculating the dose when a range shifter is used.^41^ During commissioning of the APB RS 6.2 and the MC RS 6.0 algorithms for clinical use, we measured the CAX depth dose for a typical breast treatment beam with a Markus parallel plate ionization chamber in a water tank for a zero‐degree gantry angle. We used different air gaps between the range shifter and the water surface. The deficiency in APB calculations was confirmed as can be seen in Fig. 3. It is clear from the data in Fig. 3 that the MC algorithm reduces the uncertainty with airgap to a clinically insignificant level. To minimize this airgap effect of the APB dose engine, we kept the airgaps as small as possible in this study.

**Figure 4:**
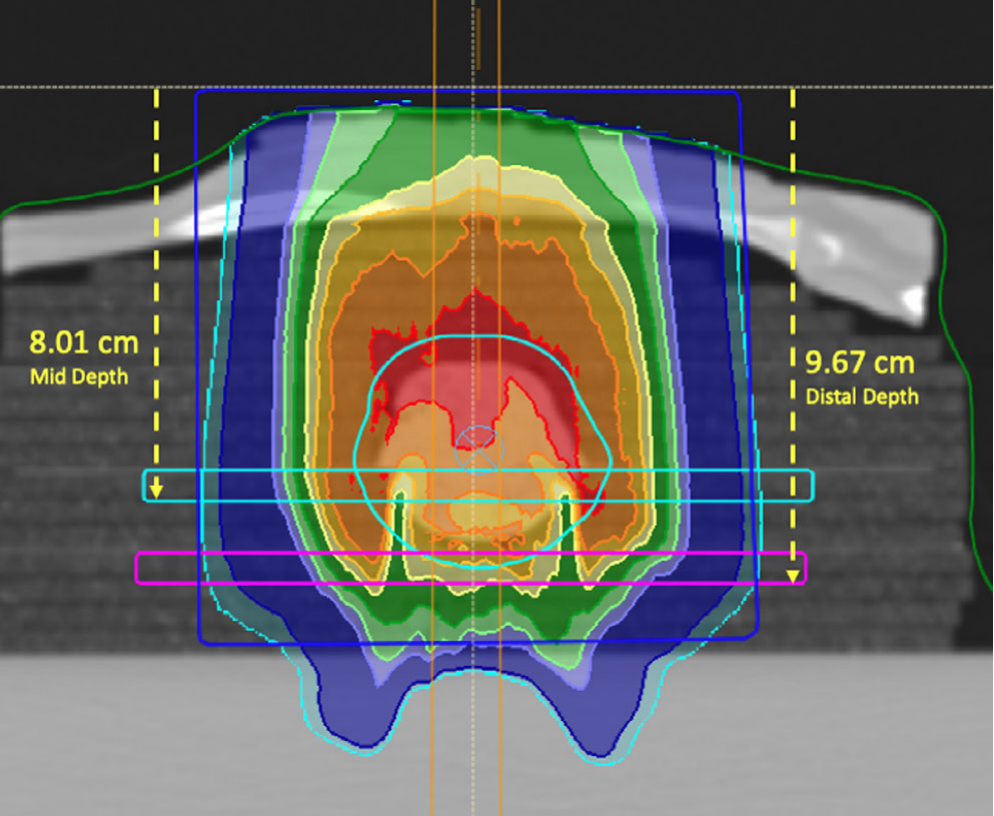
Measurement depths illustrated for verifying the calculated dose for the Anterior 1 field plan. The MatriXX PT rectangular slab contours for the mid and the distal measurement planes are illustrated with the teal and violet contours respectively. The dark blue rectangular contour shows the volume used to calculate the HU histogram shown in Fig. 5.

The MatriXX PT detector (Ion Beam Applications S.A., Louvain‐la‐Neuve, Belgium) used to measure the dose in the lung phantom has a 6.2 mm water‐equivalent buildup region proximal to the plane of measurement. This build‐up region was included in our treatment plans by drawing two 6.2 × 80 mm^2^ rectangular slab contours on each axial CT slice within the treatment volume. The top edge of each contour was aligned with the mid and distal measurement planes, respectively, that is, the positions in the phantom where the proximal surface of the MatriXX PT detector was located during the respective measurements. We simulated the insertion of the MatriXX PT detector by overriding the material in the 6.2 mm rectangular slab contours, referred to hereafter as “the MatriXX PT slab”, to water. The dose was recalculated for each of the measurement conditions at the mid and distal planes. When the mid plane dose was calculated, the MatriXX PT slab in the middle of the tumor was set to water, while the material for the distal MatriXX PT slab was not overridden. The opposite was true for when distal plane dose was calculated. The mid plane MatriXX PT slab contour (teal contour) and the distal plane MatriXX PT slab contour (purple contour) are shown in Fig. 4.

The water equivalent thickness (WET) or Relative Stopping Power Ratio (RSP) of the cork sheets was measured using 4 beam energies – 211, 195, 173 and 116 MeV respectively. Stacks of 6, 8, 16, and 18 cork sheets were placed in the beam and the resultant shift in the distal 80% dose point on the Bragg peak was measured using the IBA Zebra Bragg peak detector (Ion Beam Applications S.A., Louvain‐la‐Neuve, Belgium). A value of 0.296 ± 0.02 was obtained, i.e. a 1 cm thick cork slab will reduce the proton beam range by 0.296 ± 0.02 cm. The relatively large uncertainty of almost 7% in the measured RSP value was mainly due to measuring the thickness of the cork slabs and makes the measured RSP too imprecise to be used directly in the dose computation.

**Figure 5:**
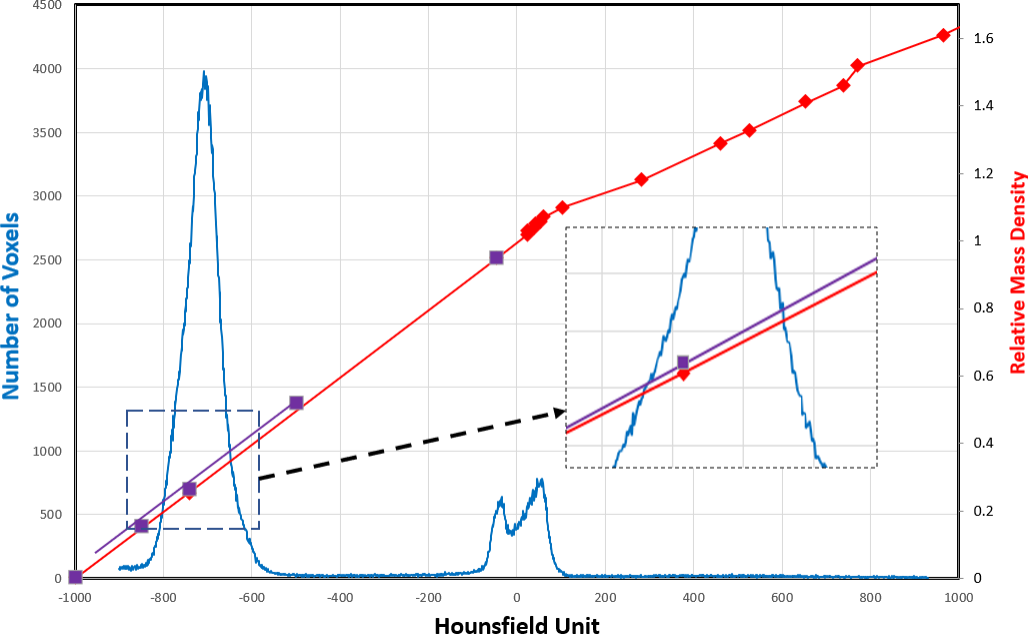
The frequency distribution of the number of voxels vs. Hounsfield unit (HU) in the calculation volume only of the lung phantom, that is, the voxels traversed by the beams, is shown by the blue line. The red line and red diamonds show the HU to Relative Mass Density calibration curve used in RayStation for routine treatment planning. The purple squares show the 5% increased mass density values in the cork region highlighted in the zoomed box.

A HU frequency distribution of the number of voxels in the volume of the lung phantom that are traversed by the beam only (see dark blue rectangular contour in Fig. 4) is shown in Fig. 5. The calculation of dose would be highly sensitive to the mass density of the cork since most voxels in the calculation volume were in the cork. The two other peaks shown in Fig. 5 are from voxels in the tumor (70% lean ground lamb meat) and the fat and soft tissue (intercostal muscle) in the rack of lamb. The original HU to mass density (HU‐to‐MD) curve, obtained using the Stoichiometric method described by Schneider et al. 42, shown in Fig. 5 (red line) gives an interpolated MD value of 0.296 (g/cm^3^) at a HU of −700, corresponding to the centroid of the cork peak in Fig. 5. According to Hünemohr et al., the MD and SPR for lung are almost equal.^43^ Our measured SPR value for cork was 0.296 ± 0.02, as mentioned earlier.

The conversion from HU to stopping power in RayStation is only expected to be correct for phantoms with human like tissue. For the present phantom the mass stopping power for cork is not expected to be the same as for lung tissue. To study the effect of the HU‐to‐MD curve a series of curves were created where the mass density of the curves was uniformly scaled by 6% up to 8% in steps of 2%. The doses of both plans were then recomputed for each of the scaled curves, and 2%/2 mm gamma analysis was conducted for the two measured planes of the two plans. Guided by these results, we recalculated all the beams with a modified HU‐to‐MD curve where the mass density was increased by 5% over the cork region only, that is, 1% more than when the entire curve was scaled, as shown in Fig. 5 (zoomed box). The 5% shift is within the uncertainty of 7% in the measured stopping power for cork, as detailed above. Because our work using animal neck phantoms and a water‐based breast phantom revealed that the HU‐to‐MD curve in the soft tissue, water and bone region is adequately accurate, we decided to leave that portion of the HU‐to‐MD curve unchanged.^19^

### 2.3 Measurements

All measurements were taken in an IBA Proteus Plus Gantry treatment room (Ion Beam Applications S.A., Louvain‐la‐Neuve, Belgium). The phantoms were aligned using external markers and the VeriSuite IGRT system (MedCom, Darmstadt, Germany), employing orthogonal x rays as performed clinically. Dose distribution measurements were taken using the MatriXX PT in the mid and distal planes only, corresponding to depths of 8.01 cm and 9.67 cm, relative to the proximal plane of the calculation grid (see Fig. 4). For the distal plane measurement at a depth of 9.67 cm, the phantom was split at the distal plane of the tumor, and only the upper section was accurately placed on the MatrriXX PT detector using the orthogonal x rays in the treatment room. The same process was repeated for the mid plane measurement at a depth of 8.01 cm, splitting the phantom at the mid plane of the tumor. In each case, the table height was adjusted to place the phantom isocenter at the beam isocenter. The toothpicks that were used to assemble the phantom ensured that the phantom was split and re‐assembled without significant shifts between the layers, maintaining the geometry of the phantom.

### 2.4 Data analysis

The MatriXX PT detector is composed of 1024 small parallel plate ionization chambers spaced 7.62 mm apart in a rectangular grid pattern. Each ion chamber has a collecting diameter of 5 mm, but were treated as dose points in the subsequent analysis. Three‐dimensional gamma analyses were performed using tools in the RaySearch Laboratories dose engine validation test suite. The gamma evaluation algorithm of this RaySearch internal software package is the same as that used in the patient specific QA system Compass© from IBA Dosimetry and RaySearch Laboratories. The measured lateral dose maps were compared to calculated APB and MC 3D dose cubes using gamma analysis parameters of 2%/2 mm, 3%/3 mm, and 5%/3 mm.^44^ The measured dose distributions were used as the reference dose distributions for the gamma index analyses. The gamma analysis was performed using absolute doses and global gamma was considered. The normalization dose was taken as the maximum dose of the computed doses.^44^ Furthermore, a 10% gamma threshold was used, that is, only doses above 10% of the max dose were included in the analyses. Prior to the gamma analysis, the measured and calculated dose planes were registered to each other to eliminate unavoidable setup errors due to the inherent resolution of the image guidance system used to align the phantom in the beam.

Using the gamma index as the only parameter to evaluate a dose calculation algorithm against measured data is perhaps not the best method, particularly for smaller fields. Since the shape of the beam might not necessarily be impacted by the tumor or inhomogeneities inside the field, most passing voxels might lie along the beam edge. Therefore, having larger discrepancies in the center of the field could still result in reasonable gamma pass rates. Extracting transverse and depth dose profiles from the calculated and measured dose planes gives quantitative dose information and provides another way to compare the doses especially in regions of disagreement.

## 3 RESULTS

**Figure 6:**
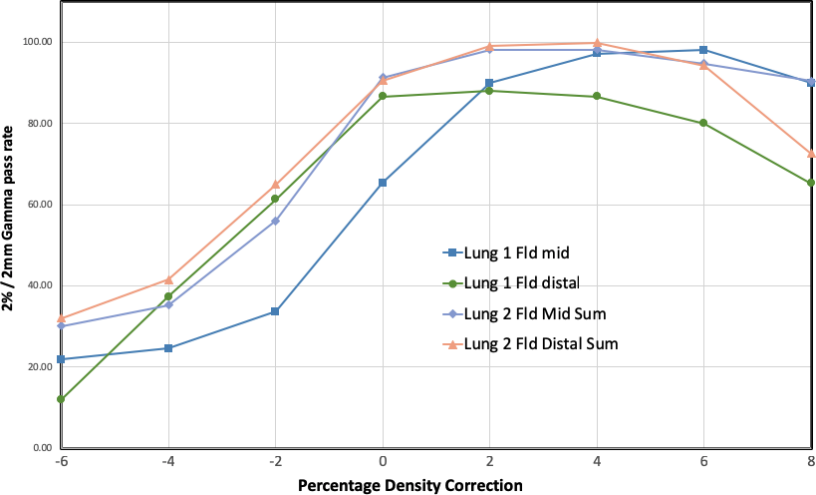
The 3D 2%/2 mm gamma pass rates for the 1 Field and 2 Field MC calculated lung plans at the expected depths as a function of the percentage correction applied to the entire HU to Mass density curve in RayStation.

The result of the 2%/2 mm gamma passing rates using the uniformly scaled HU‐to‐MD curves is depicted in Fig. 6. A maximum in passing rate is revealed for a uniform scaling somewhere between 0% and 6%, with the best average improvement for all plans and depths obtained at 4%. Three of the four cases follow very similar curves, while the Lung 1 Field plan at mid depth seems shifted about 2% with respect to the others. The reason for this slightly different behavior is unclear, but some systematic deviation in the setup of this measurement could be an explanation. We interpret the systematic improved passing rate for a uniform scaling of the HU‐to‐MD curve as mainly being caused by the difference in interpreted and the real stopping power of cork. This assumption is supported by the results in the previous study where only animal tissues were used together with the original HU‐to‐MD curve.^19^ As mentioned earlier we recalculated all the beams with a modified HU‐to‐MD curve where the mass density was increased by 5% over the cork region only. The results for the 3D gamma analyses for the calculated and measured distributions using the original and the modified HU‐to‐MD curves for both the APB and MC algorithms are tabulated in Table 1.

**Table 1:** 3D Gamma passing rates (%) comparing RayStation APB and MC doses to the MatriXX PT measurements at two depth for the two lung phantom plans using the original CT to Mass Density calibration curve as well as the calibration curve with the Mass Density scaled by 5% in the cork region.

Plan	Algorithm	Physical Plane	RaySearch 3D‐Gamma passing rate (%) @ expected depth (8.01 cm Middle and 9.67 cm distal)					
		Middle = 8.01 cm	Original Calibration Curve			Calibration scaled by 5% in Cork Region		
		Distal = 9.67 cm	2%/2 mm	3%/3 mm	5%/3 mm	2%/2 mm	3%/3 mm	5%/3 mm
Lung 1 Field	MC	Middle	65.4	92.7	95.4	83.6	98.2	99.1
	MC	Distal	86.7	92.0	92.0	86.7	92.0	92.0
	APB	Middle	43.6	71.8	80.9	68.2	84.6	92.7
	APB	Distal	78.7	85.3	85.3	82.7	86.7	86.7
Lung 2 Fields	MC	Middle	91.4	99.1	99.1	98.3	100.0	100.0
	MC	Distal	90.6	99.1	99.1	97.2	100.0	100.0
	APB	Middle	39.7	70.7	73.3	58.6	77.6	83.6
	APB	Distal	64.2	74.5	74.5	70.8	82.1	83.0

APB, analytical pencil beam.

**Figure 7:**
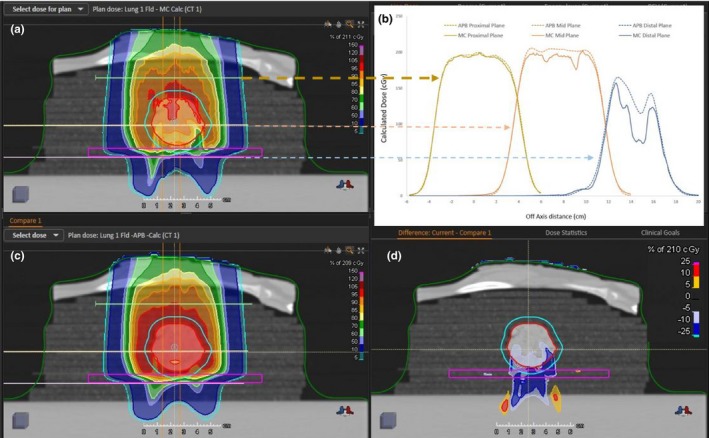
The calculated dose distributions for the single field AP beam lung plan. The Monte Carlo dose calculation is shown in panel a and the pencil beam calculation in panel c. Calculated dose profile comparisons at three different depths in the central axis plane are shown in panel b. The profiles for Monte Carlo (solid line) and analytical pencil beam algorithms (dotted line) are indicated by the yellow arrow for the proximal depth at 5.37 cm, the brown arrow for the mid depth at 7.39 cm and the blue arrow for the distal depth at 9.05 cm (expected depth = 9.65 cm). The profiles shown in panel b are offset in the horizontal axis for display purposes. Panel d shows the dose difference map between the MC and APB dose distribution in the CAX plane (MC dose minus APB dose. APB, analytical pencil beam; CAX, calculated central axis.

The MC (panel a) and APB (panel c) calculated dose distributions for the single‐field AP beam are shown in Fig. 7. Transverse profiles at three depths along the beam path are shown in panel b. The depths of the transverse profiles are indicated by the lines and the dashed arrows in panel a. The discrepancy between APB and MC dose profiles increases as the beam travels through the ground lamb meat tumor phantom and is pronounced at the edge of the tumor between the cork‐meat (lung‐tumor) interface.

**Figure 8:**
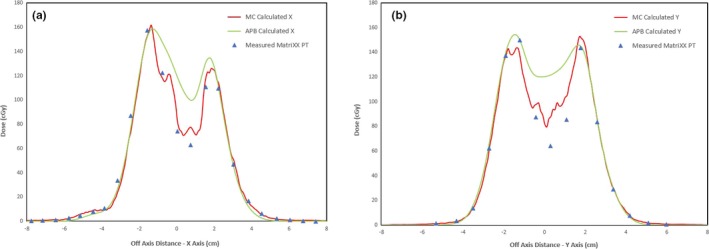
In‐Plane (panel a) and Cross‐Plane (Panel b) line dose profiles for the 1 Field lung plan at 9.65 cm depth in the dose cube. The green dots represent the APB calculated dose while the red dots are from the MC calculated dose cube. The blue triangles show the dose measured with the MatriXX PT detector. APB, analytical pencil beam.

**Figure 9:**
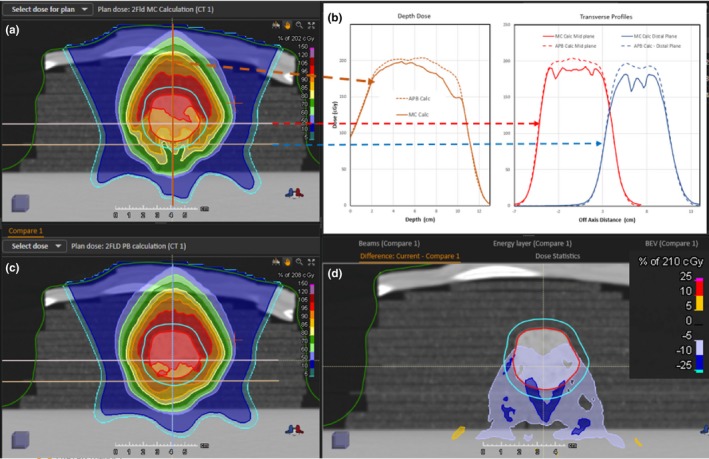
Calculated dose distributions for the two‐field beam lung plan. The Monte Carlo dose calculation is shown in panel a and the pencil beam calculation in panel c. Calculated dose profile comparisons at two different depths in the central axis plane and a depth dose comparison are shown in panel b. The profiles for the MC (solid lines) and APB algorithms (dashed lines) are indicated by the red arrow for the mid depth at 7.39 cm and the blue arrow for the distal depth at 9.05 cm. The transverse profiles are offset in the horizontal axis for display purposes. The brown arrow indicates the CAX depth dose comparison between the MC dose (solid line) and the APB dose (dotted line). Panel d shows the dose difference map between the MC and APB dose distribution in the CAX plane (MC dose minus APB dose. APB, analytical pencil beam; CAX, calculated central axis.

**Figure 10:**
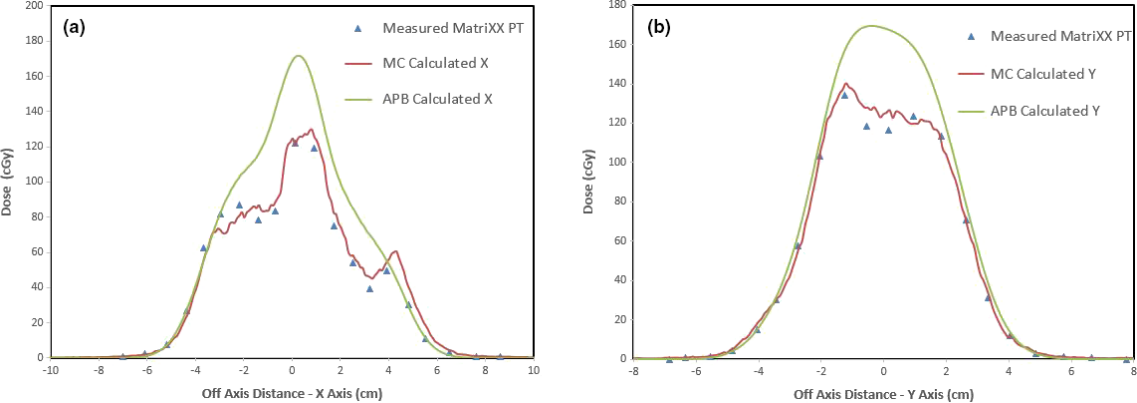
In‐Plane (panel a) and Cross‐Plane (Panel b) line dose profiles for the 2 Field lung plan at 9.65 cm depth in the dose cube. The green dots represent the APB calculated dose while the red dots are from the MC calculated dose cube. The blue triangles show the dose measured with the MatriXX PT detector. APB, analytical pencil beam.

The transverse dose profile (in‐plane x direction and cross‐plane y direction) comparisons for the measured and calculated doses in the distal plane of the single field lung plan at a depth of 9.65 cm are shown in Fig. 8. The calculated distributions for the two‐field plan are compared in Fig. 9. The transverse dose profile (in‐plane x direction and cross‐plane y direction) comparisons for the measured and calculated doses in the distal plane of the two‐field lung plan at a depth of 9.65 cm are shown in Fig. 10. There is an overestimation of dose from the APB algorithm, whereas MC predicts the measured values much more closely.

## 4 DISCUSSION

### 4.1 Interpretation of results

There is a significant overestimation of the dose in the distal part of the tumor using the APB algorithm. It deviates from the MC algorithm when the beam enters the denser‐than‐lung tumor volume. As noted by Taylor et al., APB algorithms fail here “because they fail to properly account for lateral scatter [*i.e.* lateral heterogeneities] and loss of electronic equilibrium.”^11^ This deficiency is due to the so‐called infinite‐slab‐approximation, where each pencil‐beam raytrace in the analytical dose engine sees the patient as a stack of semi‐infinite slabs.^7^, ^8^, ^9^, ^24^ The RayStation APB algorithm utilizes a sub‐spot approach where each spot is discretized into 19 sub spots to alleviate this problem. Additionally, the transport of secondary protons created in the range shifter is not properly handled in the APB algorithm, leading to an overestimation of dose. As illustrated in Fig. 3, this effect increases with increasing airgap and is more pronounced in the proximal region of the patient. We hypothesize that the error introduced by the infinite‐slab‐approximation in the APB algorithm was the cause of the increasing discrepancy between APB and MC as the beam traversed the lung tumor. Data comparisons revealed that the measured and MC calculated doses can differ by as much as 30% in distal part of the tumor. Some of these discrepancies are illustrated by the dose profiles and the dose difference maps, shown in 7, 8, 9, 10. The dose distal to the tumor differed more than dose within the tumor, and the single‐field plan had greater discrepancy than the two‐field plan. These line profiles illustrate the marked superiority of MC over APB. We emphasize that the deficiencies of the analytical dose engine presented in this paper only addresses the APB dose engine implemented in RayStation. However, it appears likely that other implementations of pencil‐beam/infinite slab‐based algorithms for protons will exhibit similar problems.

Our phantom was designed to have the tumor suspended inside the lung tissue instead of being adjacent to the chest wall. Making a phantom with the tumor adjacent to the chest wall will make it harder to measure dose inside the tumor due to the curvature of the chest wall. We believe that both scenarios will show similar trends i.e. over estimation of the dose by the APB algorithm, since the problem with the APB algorithms stems from the infinite‐slab‐approximation, as explained above. The worst‐case scenario is expected to be where the tumor is surrounded by low density lung tissue, as this maximizes the lateral heterogeneity neglected by the pencil beam algorithm.

There is no reason to believe that a CT scan and a CT‐to‐MD curve calibrated for human‐like tissues would reveal the correct stopping power for cork, which allowed us to make a 5% correction in the cork mass density to obtain better results. The 5% correction is within the uncertainty limits of the measured RSP value for cork listed above. The main purpose of this study was to test the difference between the MC and APB algorithms in a highly non‐homogeneous region, such as a tumor suspended completely within the lung. The fact that we achieved extremely good depth agreements between the measured and calculated dose distributions is encouraging from a beam range accuracy perspective. However, the main finding of this work is evident in the excellent agreement in the line dose profiles between the measured and MC‐calculated distributions distal to the tumor as illustrated in Fig. 8 and Fig. 10. This was not the case for the APB calculated distributions.

### 4.2 Clinical impact of our phantom

**Figure 11:**
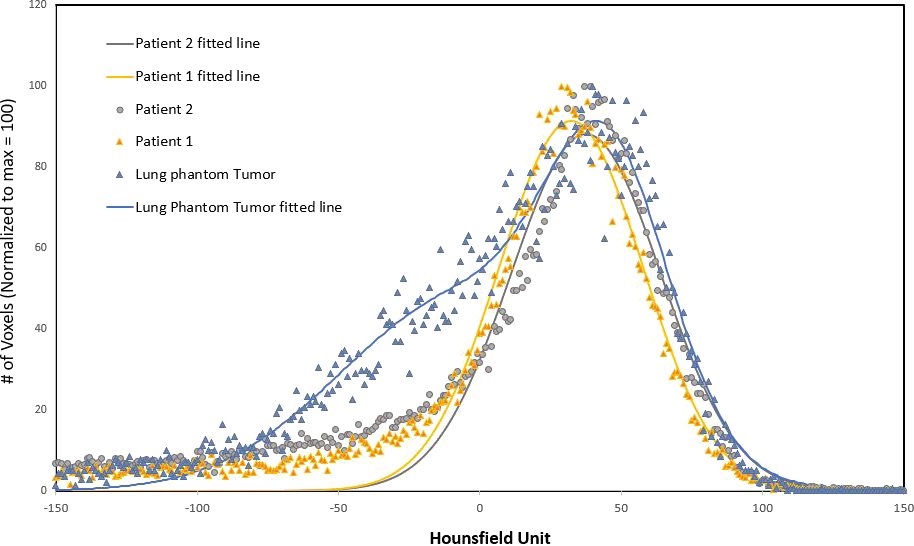
Frequency distributions of the normalized number of voxels having a certain HU for two real lung tumors treated in our clinic and for the simulated lung tumor. The data is normalized to a maximum of 100 to accommodate the different volumes of the tumors evaluated.

The ground meat simulating the tumor is a reasonable representation of a real solid tumor and is readily separable to enable dose measurement inside the tumor. Lung tumors are mostly less dense than soft tissue, which was achieved by using 70% lean ground meat as the tumor. We reviewed the HUs of solid lung tumors of two patients we treated and found that the average HU for the GTVs drawn for these patients was 35 ± 26 which compared well with our simulated lung tumor having a mean HU value of 46 + 21. The frequency distributions for two real lung tumors and for the simulated lung tumor are shown in Fig. 11. The simplicity of this model may be contrasted with an elaborate lung phantom involving a water‐filled casing with porcine lung,^45^ a commercial synthetic torso,^46^, ^47^ or a commercial pig organ phantom.^48^ Users of cork‐based phantoms should be aware of potential discrepancies between their TPS and actual stopping powers.^49^

### 4.3 RaySearch warnings

RaySearch informs the user that the pencil beam scanning dose engine uses the infinite slab approximation thereby increasing error as a function of lateral inhomogeneity.^50^ We demonstrate this behavior, as shown in 7, 8, 9, 10. They strongly recommend using the Monte Carlo dose engine for final dose computation. We concur based on the results shown in this report.

## 5 CONCLUSIONS

We have created a novel phantom simulating a lung cancer tumor, representative of the typical size and location of patient cases often treated in our clinic. This phantom enabled us to determine the errors resulting from using an analytical pencil beam algorithm for lung targets. We were able to demonstrate the superiority of the Monte Carlo dose calculation algorithm for lung targets. This work also demonstrated how the infinite slab approximation used in the APB algorithm fails when a distinct lateral inhomogeneity is encountered at the distal end of an otherwise fairly uniform medium. The case represented in this phantom is, in our estimation, one of the worst cases that one would encounter in the lung: namely, the tumor is not adjacent to the rib cage but rather suspended in the lung. This represents many centrally located lung targets. In addition, the plans created for this study were not created using robust optimization, something that would have decreased the sensitivity to dose calculation error. Therefore, based on this work and supported by many other authors referenced herein, we recommend that APB algorithms should not be used for any lung targets, and that a Monte Carlo based algorithm should be used as the dose engine for plan optimization and final dose calculation.

## CONFLICT OF INTEREST

No duality of interest disclosed.
